# Strength and Hydrophobicity of Superhydrophobic Concrete Based on Hydration Products and Surface Microstructure: Influence of Curing Temperature, Humidity, and Mesh-Coating

**DOI:** 10.3390/ma19040645

**Published:** 2026-02-07

**Authors:** Kexiao Zhou, Jie Luo, Yuan Wang, Lingyun Yang, Chenhui Chen, Wenhao Liu, Yi Xu

**Affiliations:** 1 College of Materials Science and Engineering, Hohai University, Nanjing 211100, China; 231325010017@hhu.edu.cn (K.Z.); luojiehhu@163.com (J.L.); 231325010005@hhu.edu.cn (Y.W.); 231625010083@hhu.edu.cn (L.Y.); 231625010001@hhu.edu.cn (C.C.); 2 College of Civil and Transportation Engineering, Hohai University, Nanjing 211100, China; liu1757553429@163.com

**Keywords:** superhydrophobic cement-based materials, curing regimes, microstructure, surface texture structure, contact angle

## Abstract

**Highlights:**

**What are the main findings?**

- Curing conditions optimize mechanical strength and superhydrophobicity.
- Surface texture significantly enhances superhydrophobic properties.
- Chemical stability ensures long-term performance.

**What are the implications of the main findings?**

- The standardized curing parameters for balanced performance.
- The implementation of precise texture control methods.
- The prioritization of morphology–chemistry synergy for durability.

**Abstract:**

The interplay between curing conditions and performance in superhydrophobic cementitious materials remains a critical challenge, wherein hydrophobic agent incorporation enhances hydrophobicity but often compromises mechanical strength. This study aimed to investigate the effects of curing humidity and temperature on compressive strength and contact angle and clarify the influence of surface texture on hydrophobicity. SEM–EDS, FTIR, XRD, TG, and AFM were employed to analyze the specimens. Our results showed that curing temperature positively impacts material properties, whereas excessive curing humidity enhances compressive strength but negatively affects superhydrophobicity. Additionally, micro- and nanoscale coarse structures were found to be beneficial for improving superhydrophobicity. This study offers valuable insights into the most efficient mechanism through which to optimize the preparation process for desirable properties in superhydrophobic cementitious materials.

## 1. Introduction

Due to the hydrophilic nature of cementitious materials, many corrosive media can infiltrate the interior of such materials via water transport, reducing their durability [1]. Numerous superhydrophobic surfaces exist in nature, such as rose petals, rice leaves, and lotus leaves. Water droplets can roll freely across these surfaces without being adsorbed. In-depth studies on superhydrophobic surfaces have revealed their remarkable role in the fields of self-cleaning [2,3], corrosion resistance [4,5,6,7,8,9], oil–water separation [10], directional transportation of liquids [11], and spontaneous transportation of liquids [12]. Investigating superhydrophobicity in cementitious materials can enhance their self-cleaning capabilities, corrosion resistance, and freeze–thaw resistance and extend the lifespan of structures. When the surface of superhydrophobic cementitious materials is immersed in water, a partition layer can form on the solid surface, preventing water from penetrating into the material [13]. Moreover, the water droplets on the surface of cementitious material are able to roll off the surface freely, removing the dust on the surface and improving the aesthetics of the structure.

The fabrication of superhydrophobic cementitious materials requires satisfying two essential criteria: the first is to use low surface energy substances for modification, and the second is to construct systems with rough structures. These two conditions play a significant role in the performance of superhydrophobic cementitious materials [14]. The process of superhydrophobic modification of low surface energy materials and cement hydration involves several complex reactions, and environmental factors such as temperature and humidity can significantly impact these reactions. The selection and application of low surface energy substances are important steps to realize the superhydrophobic properties of cementitious materials. Introducing low surface energy compounds, such as fluorides [15] or silanes [16], onto the surface of the material can significantly reduce its surface energy, making it strongly hydrophobic. In addition, the construction of microstructures with rough surfaces is key to enhancing hydrophobicity. By means of mechanical processing, chemical etching [17], or doping with rough particles [18,19], multi-layered rough structures can be formed on the material surface to promote superhydrophobicity.

Based on to Wenzel’s theoretical model, when the surface of a solid exhibits hydrophobicity, the hydrophobicity increases as the surface texture rougher [20,21]. Some research groups have produced superhydrophobic concrete by using silicone to reduce the surface energy and a metal mesh to build rough surface texture on the surface [22,23,24]. Wong et al. [25] produced a hydrophobic powder featuring a 153° water contact angle. Wang et al. [26] produced hydrophobic concrete and subsequently polished the concrete surface with sandpaper to obtain superhydrophobic concrete exhibiting a 153.7° contact angle. Maintenance temperature and humidity have a great influence on cementitious materials. The strength, durability, and shrinkage properties of cementitious materials vary under different temperature and humidity conditions [27,28]. Li [29] cured a superhydrophobic concrete using a curing regime of 25 ± 1 °C and 60 ± 5% relative humidity for 28 d. The maintenance temperature and humidity can significantly affect the speed and degree of cement hydration reactions. Higher temperature and humidity can promote the hydration of cementitious materials, which in turn affects the microstructure and macroscopic properties of the material. In terms of the superhydrophobic properties of cementitious materials, the effect of maintenance temperature and humidity remains unclear. It is therefore imperative to conduct further research in this field. As detailed herein, after determining the appropriate curing conditions for superhydrophobic concrete, the hydrophobicity of the concrete under the binary synergy of hydrophobic agents and surface texture modification was investigated by altering the surface texture of the specimens using the mesh covering technique.

In this study, the effects of varying curing temperature and humidity conditions on the hydrophobicity and compressive strength were separately investigated. Subsequently, we controlled the variable surface texture of the specimen to investigate its wettability characteristics. SEM–EDS, FTIR, XRD, and TG were employed to analyze the hydration products of superhydrophobic concrete, to determine the effects of curing humidity and curing temperature on the specimens, and to more comprehensively explore how curing regimes affect superhydrophobic concrete. AFM analysis was employed to explore how the surface texture affects superhydrophobicity.

## 2. Materials and Methods

### 2.1. Materials

Portland cement from Nanjing Hailuo Cement Co., Ltd. (Nanjing, China) was used in this study. The physical properties of natural sand were as follows: 2.89 fineness Modulus; 2.66 g/cm^3^ Apparent Density; 1.54 g/cm^3^ loose bulk density; 1.69 g/cm^3^ tap density, and 8.49% void fraction. Nano silica (NS; 200 m^2^/g specific surface area, 13 nm average diameter), triethoxy-(2,4,4-trimethylpentylsilane (TETPS), and octyltrimethoxysilane (OTMS) were purchased from Hangzhou Jessica Chemical Co. (Hangzhou, China). Dodecyltrimethoxysilane (DTMS) was purchased from Nanjing U-Pro Chemical Co. (Nanjing, China). Superplasticizer (water reducing rate: 30%) and corrosion inhibitor (TIA) were produced by Sobute New Materials Co., Ltd. (Nanjing, China). The surface texture structure was constructed by utilizing stainless steel woven wire mesh with mesh sizes of 20, 40, 60, 80, 100, 150, 200, and 250 mesh. The correspondence between mesh size and pore size is shown in Table 1.

### 2.2. Mix Proportion

The water-to-cement ratio and cement-to-sand ratio were 1:2. The mix proportion is shown in Table 2. The doping amounts in the table are all mass fractions of cement.

### 2.3. Specimens Preparation

First, NC was dispersed in water and stirred for 30 s to prepare a mixed solution before the addition of the hydrophobic substances (such as DTMS and TIA) into the mixed solution, and preparation of the hydrophobic solution was achieved via ultrasonic dispersion for 30 min at 40 °C.

During molding, cementitious materials and sand were added into the mortar mixer and mixed for 1 min. Thereafter, the hydrophobic solution was added and stirred for 4 min. The slurry was then poured into the mold (40 × 40 × 40 mm cube) and demolded after 1 d. Subsequently, the specimens were cured under different curing conditions for 28 d. (During the preparation process, the curing temperature was set to 10 °C, 20 °C, 30 °C, and 40 °C; the curing humidity was set to 35%, 55%, 75%, and 95%, respectively. See in Table 3).

During the surface texture test, the mixture was poured into the mold (40 × 40 × 40 mm^3^ cube), the different mesh nylon nets were attached to the surface immediately, and demolding was performed after 1 d. Thereafter, curing took place under the same curing conditions.

### 2.4. Test

#### 2.4.1. Wettability Test

The water contact angle (WCA) was determined using a contact angle meter (DSA30, Kruess, Hamburg, Germany) at 20 ± 5 °C to identify the hydrophobic properties. The probing liquids volumes were approximately 5 μL for the WCA test. Each WCA value was the arithmetic mean of five independent measurements taken at different locations on one specimen. The droplet images were captured using the WCA measuring instrument. A WCA tester was used to measure the rolling angle (SA) using 18 μL of water droplets at room temperature, measurements were performed in triplicate, and the arithmetic mean was reported as the final result.

#### 2.4.2. Compressive Strength Test

The loading rate was controlled at 2.4 KN/s ± 0.2 KN/s during the strength test, the strength of ordinary cement-based materials and specimens with the addition of hydrophobic substances was measured on a 40 mm^3^ cube for 28 days. Results were determined by calculating the arithmetic mean of three replicate specimens.

#### 2.4.3. Microstructural Analysis

The surface morphology of the samples was examined using a scanning electron microscope (SEM, ZEISS Sigma 300, Germany). The crystalline phase composition was determined by X-ray diffraction (XRD) using a X-ray powderdiffractometer (Rigaku -D/max 2200pc, Japan) with Cu Kα radiation (λ = 1.54 Å). The patterns were recorded over a 2θ range of 5° to 90° at a scanning speed of 2°/min. TG analysis was conducted to evaluate the change rule of the specimen hydration product. For this test, we utilized the Japan Rigaku TG/DTA8122 instrument, the gas atmosphere in the test process is nitrogen, the temperature is 30~1000 °C, and the heating rate is 20 °C/min. FTIR was employed to characterize the functional groups present in the materials and to characterize the changes therein. The testing instrument employed was the Thermo Scientific Nicolet iS20 (Waltham, MA, USA), and the testing mode was powder conventional pressing with a wave number range of 400~4000 cm^−1^.

## 3. Results and Discussion

### 3.1. Effect of Curing Humidity

**Figure 1:**
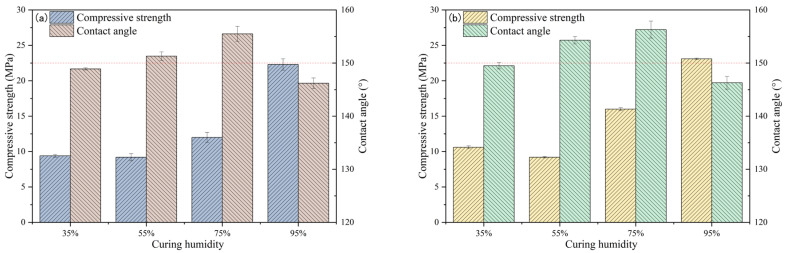
Effect of curing humidity on compressive strength and contact angle of DT2A10 (**a**) and OT2A10 (**b**).

The dependence of strength on the curing humidity and contact angle of DT2A10 is illustrated in Figure 1a and Table 4; when the maintenance humidity is 35% and 55%, the strength of DT2A10 is comparable; when the maintenance humidity is more than 55%, the strength increases with increasing humidity. Compressive strength increases to 22.3 MPa when the humidity is 95%. With the elevation of maintenance humidity, DT2A10’s contact angle showed a trend of increasing and then decreasing; the contact angle reached the highest value of 155.5° under 75% humidity, and the contact angle decreased to 146.2° under 95% humidity. The effect of maintenance humidity on the strength and hydrophobicity of OT2A10 is graphically represented in Figure 1b; when the maintenance humidity is more than 55%, the strength increases continuously with the increase in the maintenance humidity, and the compressive strength is 23.1 MPa under 95% humidity. The hydrophobicity of OT2A10 also exhibited an initial increase followed by a decrease with increasing curing humidity, and the contact angle reached the maximum value of 156.3° under 75% humidity, whereas the contact angle decreased to 146.3° under 95% humidity. Our results illustrate the hydrophobicity and strength of the specimens that can be realized when the maintenance humidity is set at 75%.

**Figure 2:**
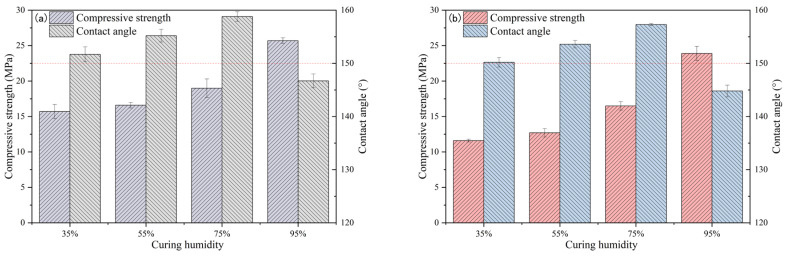
Effect of curing humidity on compressive strength and contact angle of TE4A6 (**a**) and TE2A10 (**b**).

The influence of curing humidity on the compressive strength and contact angle of TE4A6 is presented in Figure 2a and Table 5; with an increase in curing humidity, the strength of TE4A6 gradually increased, and when the relative humidity during curing was 95%, the compressive strength reached 25.7 MPa, an increase of 62.7% compared with the results obtained with a curing humidity of 35%. Higher relative humidity facilitated the hydration of cementitious materials, leading to a gradual enhancement of strength. With an increase in humidity, the contact angle of TE4A6 showed a tendency of increasing and then decreasing, reaching a maximum value of 157.4° when the humidity was 75% and decreasing to 146.7° when the humidity was 95%. This finding is related to the modification process of TETPS, which involves a series of reactions; increased humidity promotes the hydrolysis reaction of TETPS, which subsequently undergoes dehydration condensation with hydroxyl groups in the mortar. This reaction grafts hydrophobic alkyl groups (-CH_3_ and -CH_2_) onto the surface of the hydration products, resulting in a continuous increase in the contact angle. When the humidity reached 95%, the excessive humidity may have affected the dehydration–condensation process, leading to a decrease in the contact angle. The effect of maintenance humidity on the strength and contact angle of TE2A10 is shown in Figure 2b; the strength of TE2A10 was comparable when the humidity was 35% and 55%; when the humidity was more than 55%, the strength increased gradually with the increase in humidity, and when the humidity was 95%, the compressive strength was 24.0 MPa. This finding may be explained by the fact that, under lower-humidity conditions, the hydration process of cement is mainly controlled by water when added during the mixing process, and the low humidity environment has a limited promotion effect on cement hydration reaction. When the humidity is higher, the water molecules in the environment can influence the process of hydration more effectively. The contact angle of TE2A10 reached a maximum value of 157.7° when the humidity was 75% and decreased to 144.9° when the humidity was 95%.

### 3.2. Effect of Curing Temperature

**Figure 3:**
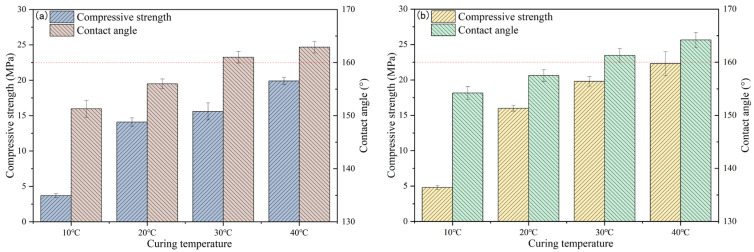
Effect of curing temperature on compressive strength and contact angle of DT2A10 (**a**) and OT2A10 (**b**).

Figure 3a and Table 6 illustrates the influence of curing temperature on the compressive strength and contact angle of DT2A10; with increasing curing temperature, the strength of DT2A10 exhibits a continuous upward trend. At a curing temperature of 10 °C, the compressive strength of DT2A10 was a mere 3.7 MPa, and when the curing temperature was increased to 40 °C, the compressive strength increased to 19.9 MPa, a 437.8% increase compared to the strength recorded at 10 °C. The contact angle of DT2A10 also exhibited a progressive increase with rising curing temperature, with a contact angle of 151.3° at 10 °C and an increase to 162.9° when the curing temperature was raised to 40 °C. The contact angle of DT2A10 was also increased with the increase in curing temperature. As depicted in Figure 3b, the performance of OT2A10 followed a similar pattern, whereby its strength gradually increased with temperature. When the temperature was 10 °C, the compressive strength of OT2A10 was only 4.8 MPa; in comparison, when the temperature was 40 °C, the compressive strength reached 22.3 MPa, an improvement of 364.6%. Moreover, the contact angle of OT2A10 increased from 154.2° to 164.2°. Notably, under identical curing conditions, OT2A10 demonstrated better performance compared to that of DT2A10.

**Figure 4:**
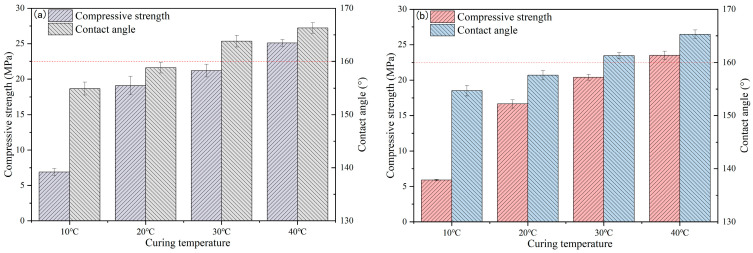
Effect of curing temperature on compressive strength and contact angle of TE4A6 (**a**) and TE2A10 (**b**).

The effect of curing temperature on the compressive strength and contact angle of TE4A6 is shown in Figure 4a and Table 7, which shows a gradual rise in its strength with the increase in curing temperature. When the temperature was 10 °C, the compressive strength was a mere 6.9 MPa; in comparison, when the temperature was raised to 40 °C, the compressive strength increased significantly to 25.1 MPa, an increase of 263.8%. Increased curing temperatures exert a substantial positive influence on strength enhancement by effectively catalyzing cement hydration kinetics It has been shown [30,31] that an increase in curing temperature aids in accelerating the dissolution and initial hydration of the cement and accelerating the rate of hydration product generation. The hydrophobicity of TE4A6 showed a gradual increase under rising thermal conditions. At a curing temperature of 10 °C, the contact angle of the specimen exceeded 150° and reached 154.9°, while the contact angle further increased to 166.3° when the curing temperature was 40 °C, showing excellent superhydrophobicity. Theoretical analysis results suggest that elevated curing temperatures amplify hydrolysis reaction velocity and increase the reaction rate constant [32]. This finding may be explained by the fact that, under high temperature conditions, the movement of molecules is more vigorous, increasing the frequency of intermolecular collisions and amplifying the possibility of chemical reactions. In addition, the increase in temperature promotes the rate of the dehydration condensation reaction. At higher temperatures, the kinetic energy of the reactant molecules increases, leading to more frequent collisions and interactions between them, which in turn enhances the reaction rate. The dehydration–condensation process is a reversible reaction, with both forward and reverse directions, and based on Le Chatelier’s principle, the increase in temperature biases the reaction towards the heat-absorbing forward process. Therefore, under higher temperature conditions, the tendency of the positive reaction is enhanced, leading to an increase in the production of products, which in turn improves the mortar’s contact angle. The effect of curing temperature on the compressive strength and contact angle of TE2A10 is shown in Figure 4b, which shows a clear increasing trend of the strength of TE2A10 with the increase in curing temperature. Under a curing regime of 10 °C, the compressive strength of TE2A10 was a mere 5.9 MPa, and when the temperature was raised to 40 °C, the compressive strength increased significantly to 23.5 MPa, a value 298.3% higher than that at the 10 °C curing temperature. The hydrophobicity of TE2A10 also showed an increasing trend with the increase in curing temperature, and the contact angle was greater than 150° at the 10 °C curing temperature, i.e., 154.7°, and at 40 °C, the contact angle further increased to 165.3°. The obtained data met expectations (at 75% humidity, cement hydration can proceed sufficiently, while promoting the hydrolysis of hydrophobic agents without causing the loss of hydrophobic functional groups), demonstrating excellent superhydrophobicity with contact angles higher than those reported in previous studies (e.g., 153° [25] and 153.7° [26]).

Despite the difference in dosage between TE4A6 and TE2A10, the effect of curing temperature on strength and contact angle showed an identical trend for both. Under the same curing temperature conditions, TE4A6 exhibited better performance compared to that of TE2A10. Taking strength and contact angle as the evaluation criteria, the modification effects of the four composite hydrophobic agents on mortar preparation were, in descending order, TE4A6, TE2A10, OT2A10, and DT2A10.

### 3.3. Surface Texture Structure

**Figure 5:**
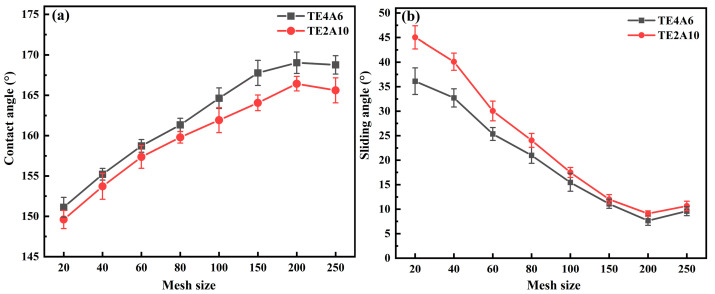
Effect of surface texture structure on (**a**) contact angle and (**b**) rolling angle.

Figure 5a and Table 8 illustrates the dependency of contact angles (CAs) for TE4A6 and TE2A10 on surface texture, influenced by the stainless steel mesh count; as the mesh number increases, the contact angle gradually rises, and when the mesh size is 80 mesh (0.21 mm), the contact angle of TE4A6 reaches 161.3°. When the mesh numbers are 200 mesh (0.08 mm) and 250 mesh (0.05 mm), the change in the contact angle of TE4A6 is relatively stable, remaining at roughly 169°. For TE2A10, the contact angle reaches 161.9° at 100 mesh (0.18 mm). Upon reaching higher mesh counts of 200 (0.08 mm) and 250 (0.05 mm), the CA values plateaued, stabilizing at approximately 166°. This finding indicates that the surface texture structure effectively enhances the hydrophobicity of the specimens. When the mesh number is 200 and 250 mesh, the roughness of the specimen surface tends to be comparable; therefore, the trend of the contact angle is relatively small. The effect of the surface texture structure on the rolling angle is shown in Figure 5b; the rolling angle of the specimen gradually decreases with the increase in mesh number. With a 20 mesh size (1.05 mm), the rolling angle exceeded 30°. Our results showed that the rough structure at this scale did not reach the micro-nanometer level, resulting in the wetting state of the water droplets similar to the Wenzel state, which exhibited a high rolling angle. When the mesh size was increased to 100, the rolling angle of TE4A6 and TE2A10 decreased to about 15°. Our results showed that, as the scale of the surface texture structure gradually approaches the micro-nanometer level, the wetting state of the water droplets gradually transitions from the Wenzel state to the Cassie–Baxter state, resulting in a gradual decrease in the rolling angle of the specimens. When the mesh size reaches 200 (0.08 mm), the rolling angle of TE4A6 and TE2A10 decreases to less than 10°, showing excellent superhydrophobicity with a very low rolling angle. At this scale, the specimen surfaces possess a good micro- and nanoscale structure. With the further increase in mesh size to 250 (0.05 mm), the rolling angle increased slightly. It was concluded that this mesh number may be too large, impacting the formation of the surface texture structure and reducing the texture of the specimen surface.

### 3.4. SEM Analysis

**Figure 6:**
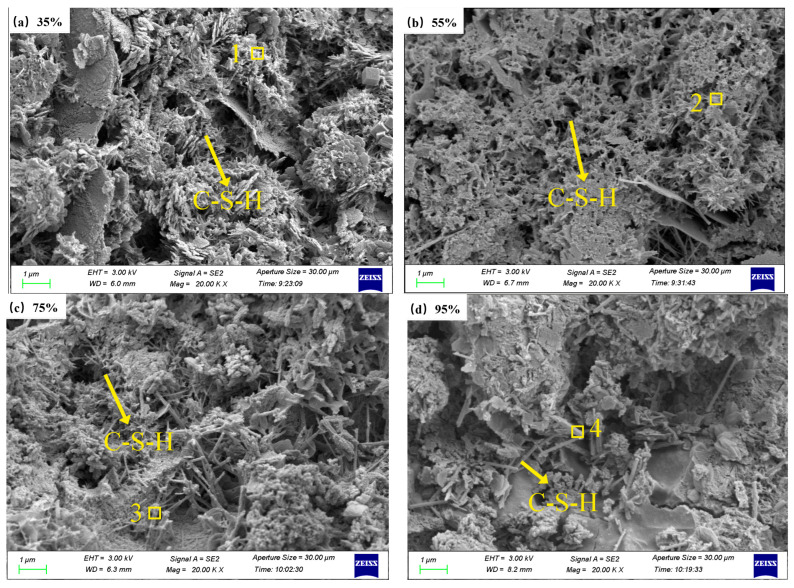
SEM photos and EDS images of TE4A6 at different curing humidities.

SEM images derived from specimens subjected to varying curing humidities are shown in Figure 6. The micro-morphology of the specimens at different conservation humidities exhibits variability. The main hydration products can be observed in the micro-morphology, in which the needle- and rod-like products may be ettringite, and the fibrous, networked or wrinkled foil-like products may be C-S-H gels. The degree of hydration of the cement was gradually enhanced with the increase in the maintenance humidity. When the humidity is 35%, the hydration degree of the cement is relatively low, and the hydration products are not effectively cross-linked to form an overall structure, showing a loose shape. When the humidity is 55%, the hydration degree of the cement is enhanced, the hydration products increase, and a number of C-S-H gels cross-link with each other to form a whole. When the humidity is 75%, the hydration products of cement are mainly fiber-like C-S-H gels. When the humidity is 95%, the maintenance conditions of cement hydration reaction are satisfied, and the hydration reaction can be completed in full, the degree of hydration exhibits an evident increase, and the structure becomes denser. This finding also demonstrates that under high-humidity conditions, the hydrophobic functional groups of silane degrade, enabling more complete cement hydration, which in turn leads to a decline in the contact angle at excessively high curing humidity levels.

**Figure 7:**
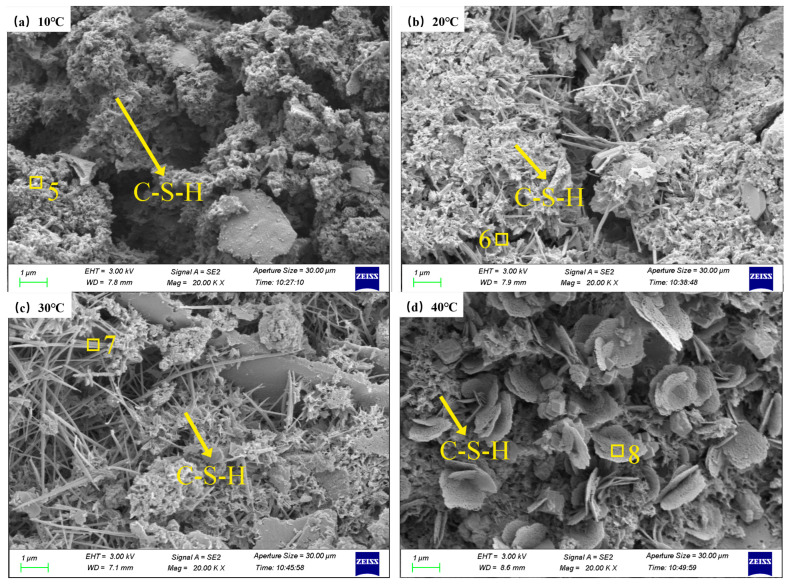
SEM photos and EDS images of TE4A6 at different curing temperatures.

The SEM micrographs of specimens subjected to varying curing temperatures are presented in Figure 7. The microscopic morphology of the specimens at different curing temperatures varied greatly, and C-S-H gels with diverse morphologies were present therein. When the temperature was 10 °C, the hydration products of the specimens were mainly network-like C-S-H gels, which could not be cross-linked into a whole and had a relatively loose structure. When the temperature was 20 °C, the hydration degree of the cement enhanced, the network-like C-S-H gel grew into a fibrous structure, and part of the network-like C-S-H gel grew on the surface of the fibrous C-S-H gel, forming a cross-linked structure, making the structure gradually denser. When the temperature was 30 °C, numerous fibrous C-S-H gels formed inside the specimen, and a number of cluster-like C-S-H gels became very tight and grew interlaced, filling the middle of the fibrous C-S-H gels, making the structure denser. When the temperature rose to 40 °C, the hydration degree of the cement increased once again with the further increase in temperature, and the fibrous C-S-H gels were connected with various hydration products, with many of the C-S-H gels becoming interlayer-like and the structure becoming denser.

### 3.5. XRD Analysis

**Figure 8:**
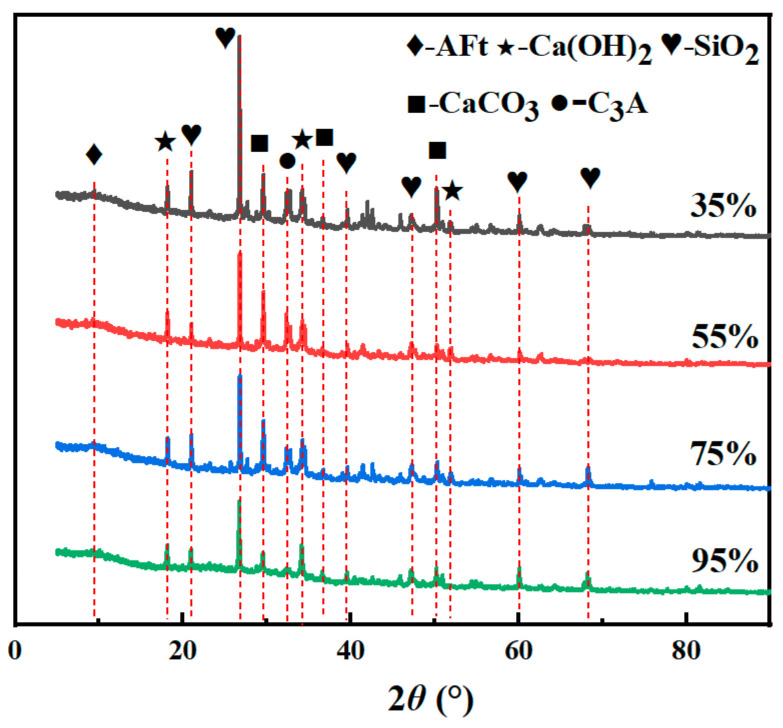
XRD patterns of TE4A6 at different curing humidities.

Figure 8 illustrates the XRD patterns of specimens cured under different humidity conditions. The change in the curing humidity does not change the type of physical phase of the specimens, and the types of hydration products are basically similar, with the main hydration products being Ca(OH)_2_ and unreacted cement clinker minerals. It can be observed that the positions of the characteristic peaks of each physical phase are fundamentally identical; however, there are differences in the intensity of the peaks. Since the molded specimens contained river sand and NS, multiple characteristic peaks of SiO_2_ can be observed, which mainly appeared at 2Theta = 21.0°, 26.8°, 39.6°, and 60.1°. In the XRD patterns, the diffraction peaks associated with Ca(OH)_2_ increase with increasing curing humidity; in comparison, peaks assigned to clinker minerals such as C_3_A show a gradual reduction in intensity. This finding suggests that clinker minerals in the cement are progressively consumed, accompanied by a continuous increase in hydration products as maintenance humidity rises. The higher maintenance humidity contributes to accelerated hydration, thereby consuming the cement clinker minerals and enhancing the yield of hydration products.

**Figure 9:**
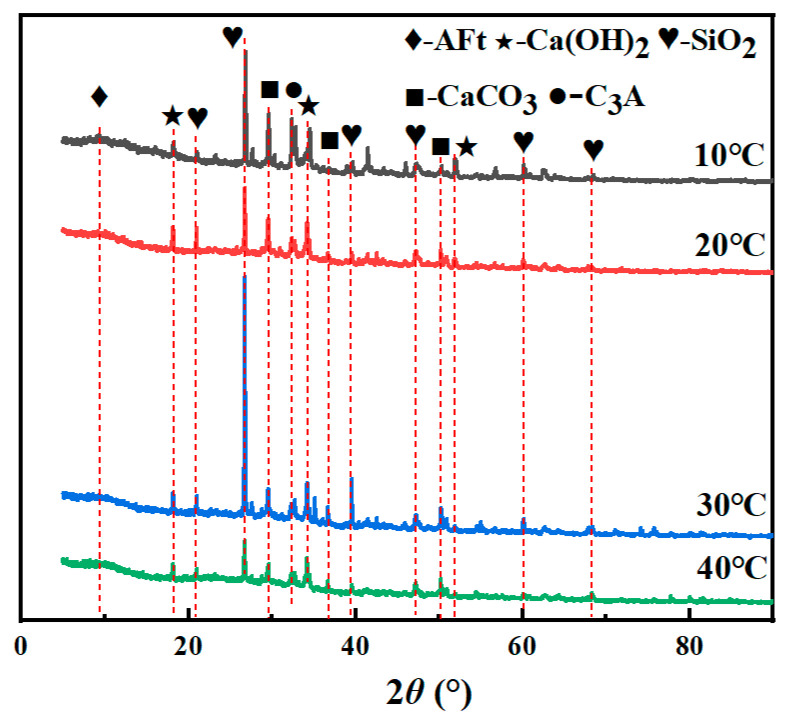
XRD patterns of TE4A6 at different curing temperatures.

The XRD patterns of the specimens under different conservation temperatures are shown in Figure 9. The change in curing temperature does not change the type of physical phase of the specimen, and the types of hydration products of the two are fundamentally similar, with the main hydration products being Ca(OH)_2_, CaCO_3_, etc. The XRD patterns of the specimens under different curing temperatures are presented in Figure 9. The characteristic peak positions of each physical phase are fundamentally identical; however, there are differences in the intensity of the peaks. As the molded specimens contain river sand and NS, multiple characteristic peaks of SiO_2_ can be observed. Diffraction peaks associated with Ca(OH)_2_ mainly appeared at 2Theta = 18.2°. In comparison, the characteristic peaks of CaCO_3_ mainly appeared at 2Theta = 29.6°. In the XRD patterns, the diffraction peaks associated with Ca(OH)_2_ progressively increased with the increase in curing temperature; in contrast, the diffraction signals related to clinker minerals such as C_3_A gradually decreased. This finding indicates that the clinker minerals in the cement are continuously consumed, and the hydration products increase as the curing temperature rises. An increase in curing temperature accelerates cement dissolution and early-stage hydration, thereby promoting the formation of hydration products.

### 3.6. TG Analysis

**Figure 10:**
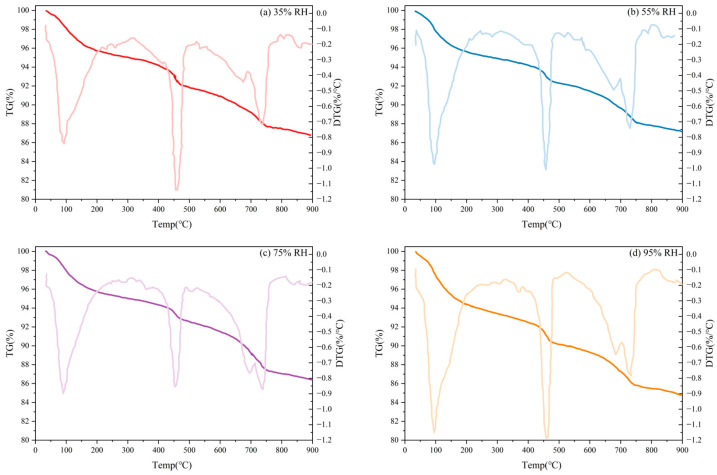
TG and DTG curves of TE4A6 with different curing humidities: (**a**) 35%RH, (**b**) 55%RH, (**c**) 75%RH, (**d**) 95%RH.

The TG diagrams of the specimens under different curing humidities are shown in Figure 10a. Specimens cured under different humidity conditions exhibited mass loss in the temperature ranges of 50–200 °C, 400–500 °C, and 600–800 °C, which mainly involved dehydration and decarburization weight loss of some hydration products. In the DTG plot presented in Figure 10b, the mass loss phenomenon of hydration products can also be observed in a similar temperature region; in comparison, the mass-loss rate differs.

**Figure 11:**
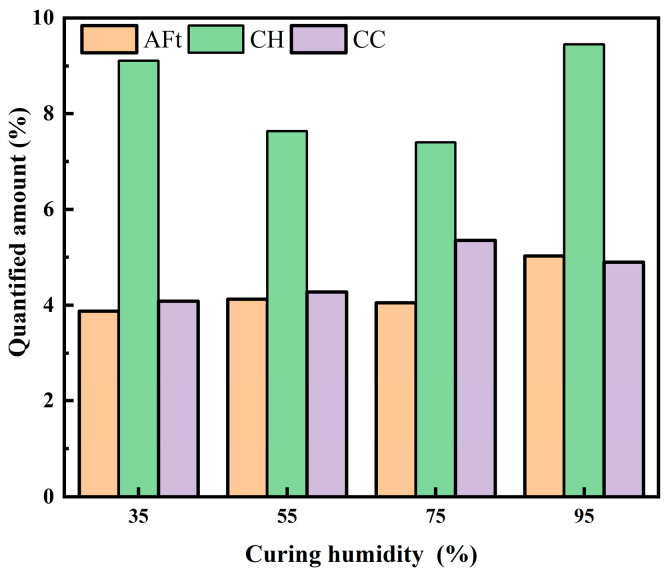
AFt, CH, and CC contents of TE4A6 with different curing humidities.

Figure 11 shows the contents of ettringite (AFt), calcium hydroxide (CH), and calcium carbonate (CC) under different curing humidities. The content of AFt gradually increased with curing humidity, and when the curing humidity was 95%, a pronounced increase in AFt content was observed. In contrast, the CH content showed an initial decrease followed by a subsequent increase; in comparison, the CC content exhibited the opposite trend, characterized by an initial increase and a subsequent decrease. Our results showed that the hydration degree of cement gradually increased with the increase in curing humidity, which led to a gradual increase in AFt content. The increase in maintenance humidity promotes the generation of CH, which in turn promotes the reaction between NS and CH and consumes CH. Therefore, the content of CH decreases when the humidity is 55% and 75%; in comparison, when the humidity is 95%, the cement exhibits a high degree of hydration, and a large amount of CH is generated, and despite the fact that part of the CH is consumed, its content is still high. The increase in maintenance humidity increases the content of CH, which carbonates to produce CC, and therefore, the content of CC gradually increases. It was demonstrated in one study [33] that humidities of 55% to 75% promote carbonization. Therefore, when the conservation humidity was 95%, the content of CC decreased. This finding can be explained by the fact that high-humidity conditions prevent carbonization from proceeding.

**Figure 12:**
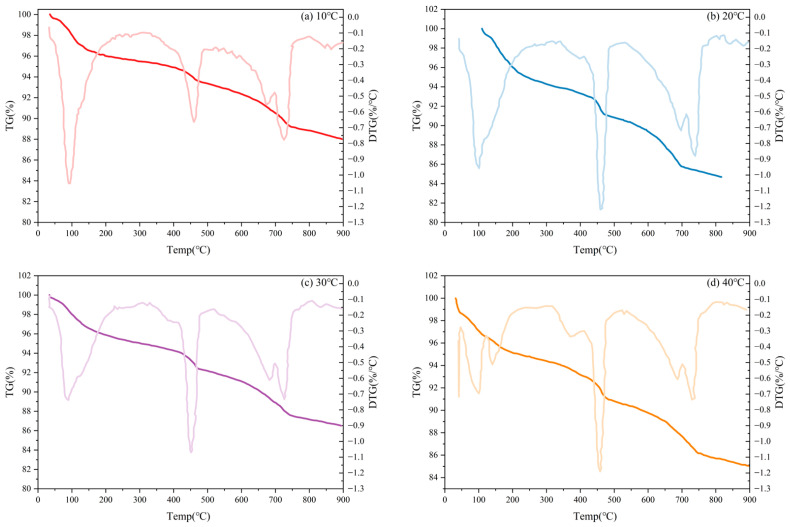
TG and DTG curves of TE4A6 with different curing temperatures: (**a**) 10 °C, (**b**) 20 °C, (**c**) 30 °C, (**d**) 40 °C.

The TG and DTG curves of specimens under different curing temperatures are shown in Figure 12, and it was found that they exhibit a similar change law, with all of them exhibiting mass loss in the same temperature interval, but with differences in the rate of mass loss.

**Figure 13:**
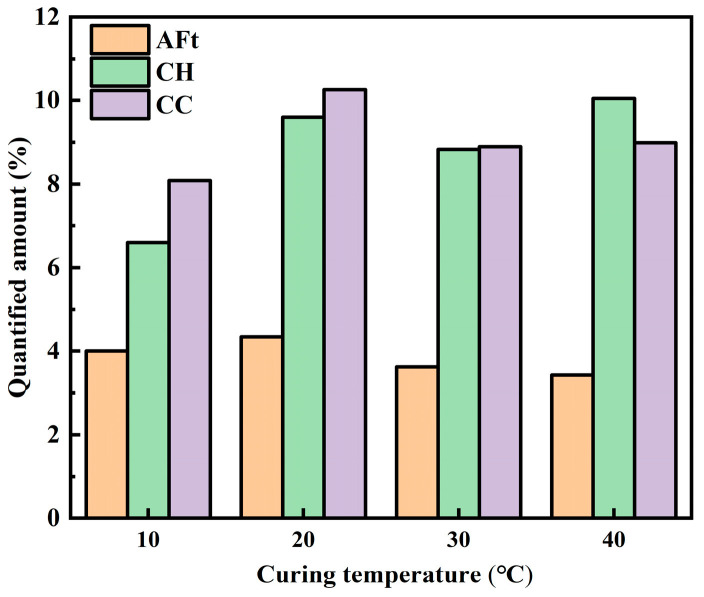
AFt, CH, and CC contents of TE4A6 with different curing temperatures.

The contents of AFt, CH, and CC under different curing temperatures are shown in Figure 13. It can be seen that, as the temperature rises, AFt content shows an initial increase followed by a subsequent decrease; CH content increases initially, then decreases, and subsequently rises again, with the maximum occurring at 40 °C; CC content follows a similar pattern and reaches its highest value at 20 °C. It can be inferred that higher temperature promotes cement hydration, thereby increasing AFt generation; however, a large amount of AFt will form a layer on the surface of C_3_A, hindering its further reaction, which reduces Aft generation. With the increase in temperature, NS and CH react with each other; thus, the generation of CH will decrease at 30 °C. With higher temperatures, however, cement hydration is further enhanced, which promotes CH formation. The change in temperature disrupts the equilibrium between water vapor and pore solution, and higher temperature leads to higher water vapor content in the pores and accelerates the movement of CO_2_ [34]. Therefore, the content of CC increases with increasing temperature, and when the temperature is 30 °C, the content of CH constrains the carbonation reaction, resulting in the yield of CC decreasing; in comparison, when the temperature rises to 40 °C, CO_2_ diffuses more easily into the interior of the cement and reacts with a greater amount of CH, increasing the yield of CC.

### 3.7. FTIR Analysis

**Figure 14:**
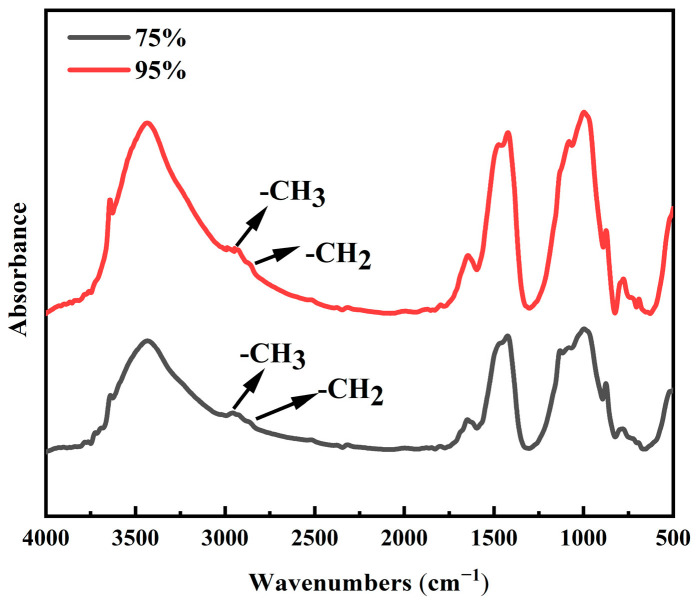
FTIR patterns of TE4A6 at different curing humidities.

The FTIR test results for TE4A6 at different conservation humidities are shown in Figure 14, wherein the functional groups of -CH_3_ and -CH_2_ are observed at both conservation humidity conditions. When the humidity was 75%, the characteristic peak of -CH_3_ was observed at 2961 cm^−1^ and that of -CH_2_ was observed at 2862 cm^−1^. When the humidity is 95%, the same two characteristic peaks are observed at the two adjacent positions; however, the intensity is lower than the intensity of the characteristic peaks when the humidity is 75%. This finding indicates that the change in humidity has a marginal effect on the dehydration–condensation process of the hydrophobic agent; however, it does not completely prevent the hydrophobic modification effect and only reduces it. Therefore, in the previous test, when the humidity was 95%, the contact angle of the specimen remained at 144.9° and did not drop below 90°, which indicates that the hydrophobic modification is still effective. The high-humidity condition adversely impacted the dehydration–condensation process of the hydrophobic substances, mainly promoting the reverse reaction of the said reaction. Therefore, a humidity of 75% is more favorable to the dehydration–condensation process of the hydrophobic agent, which is conducive to improving the superhydrophobicity of the specimens.

**Figure 15:**
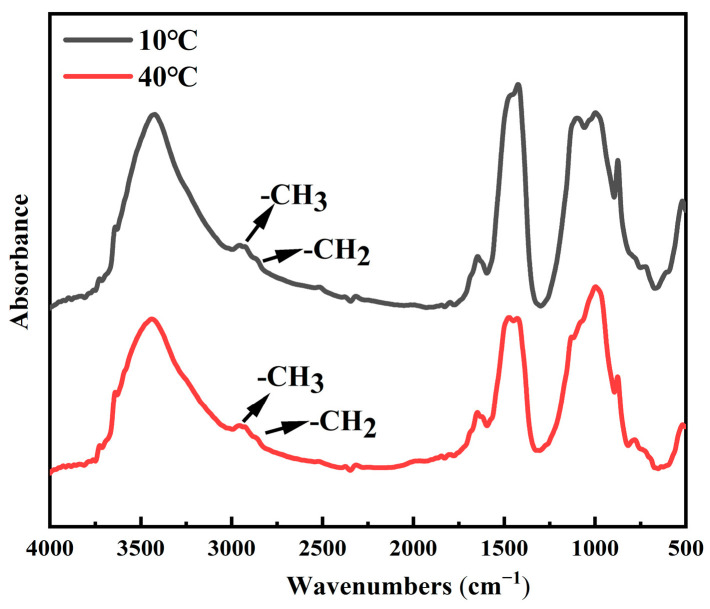
FTIR patterns of TE4A6 at different curing temperatures.

The FTIR spectra of TE4A6 obtained at various curing temperatures are presented in Figure 15, wherein the functional groups of -CH_3_ and -CH_2_ are observed under both curing temperature conditions. The increase in temperature will make the dehydration and condensation process favor the positive process of heat absorption, and the higher the temperature, the more favorable the reaction, which is conducive to the improvement of the superhydrophobicity of the specimens.

### 3.8. AFM Analysis

**Figure 16:**
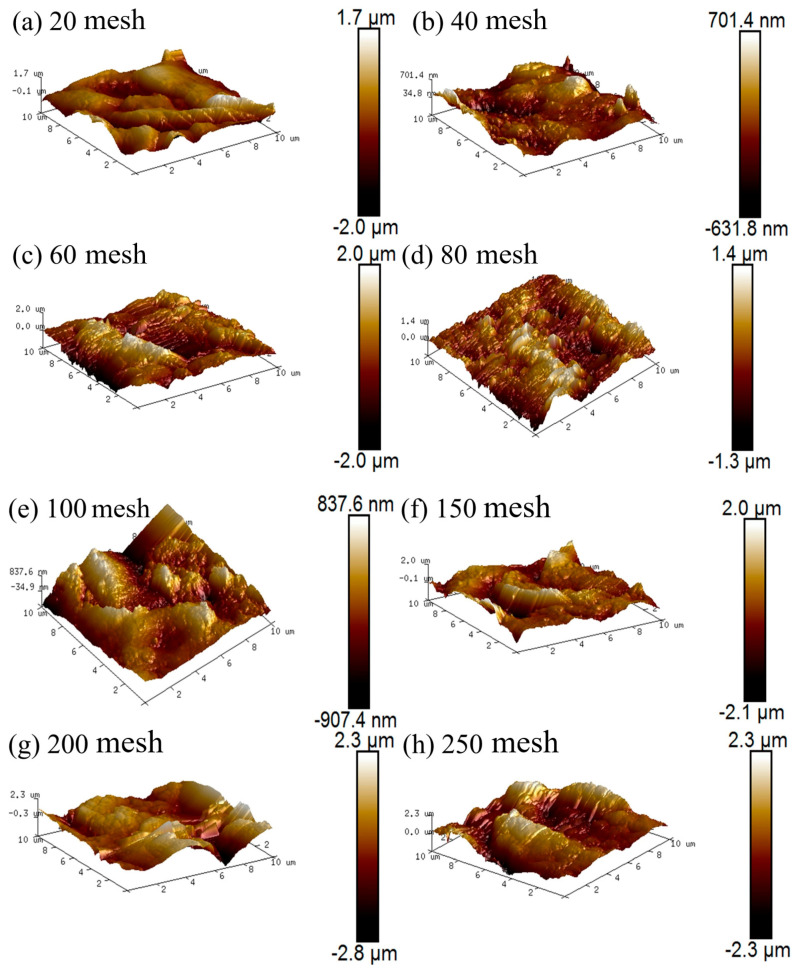
AFM plots of different surfaces.

The AFM test results for different rough structures are presented in Figure 16. For each texture level, a region on the specimen surface with uniform texture and free from visible contaminants is selected for testing. The undulations on the surface of the specimen are larger when the mesh size is 20 mesh (1.05 mm), 60 mesh (0.30 mm), and 80 mesh (0.21 mm), and the undulations on the surface of the specimen are smaller when the mesh size is 40 mesh (0.48 mm). Our results showed that the rough structure constructed using 20 mesh, 40 mesh, 60 mesh, and 80 mesh stainless steel mesh has a larger scale, and in some cases, the undulation of the surface rough structure could not be fully scanned at the microscopic angle (10 μm × 10 μm), resulting in a large difference in the undulation of the surface of the specimen. The texture of the specimen at 20 mesh reaches 383 nm. At 40 mesh, the texture of the specimen is 170 nm, and the surface is relatively smooth. At 60 mesh, the texture of the specimen surface is 277 nm, indicating a relatively rough surface. When the mesh number is increased to 80, the texture of the specimen surface further increased to 310 nm. Due to the large spacing of the surface texture of these stainless steel mesh structures with varying mesh numbers, the water droplets in the wet state on the surface are still close to the Wenzel state, resulting in a high rolling angle on the specimen surface [35]. When the mesh number is 100 mesh (0.18 mm), the undulation of the specimen surface ranges from −907 nm to 837 nm, with a texture of 240 nm. Our analysis results suggest that the wetting state of the water droplets gradually transitions from the Wenzel model to the Cassie–Baxter model as the scale of the rough structures approaches the micro-nanometer scale [36]. When the mesh size is 150 mesh (0.11 mm), the undulation of the specimen surface ranges from −2.1 μm to 2.0 μm, and the texture is 527 nm, which indicates that the texture of the specimen surface improves with the gradual refinement of the texture structure, and the wetting state of the specimen surface is closer to the Cassie–Baxter model. When the mesh size is 200 mesh (0.08 mm), the undulation of the specimen surface ranges from −2.8 μm to 2.3 μm, and the texture is 639 nm, which shows that the undulation and texture of the specimen surface have been improved, and the wetting state is in conformity with the Cassie–Baxter model, which indicates that the specimen possesses a very low rolling angle. When the mesh size is 250 mesh (0.05 mm), the undulations of the specimen surface range from −2.3 μm to 2.3 μm, and the texture is 613 nm. Compared with that of 200 mesh, the undulations and texture of the specimen surface decreased, which was consistent with the previous results showing the increase in rolling angle at 250 mesh.

## 4. Conclusions

Combining the preparation factors of cementitious materials with superhydrophobicity and the actual construction background, the effects of curing temperature and curing humidity on the compressive strength and contact angle of mortar, as well as the effects of surface texture structure on the contact angle and rolling angle of materials, were studied, leading to the following conclusions:

(1) The specimen compressive strength gradually increased as curing humidity rose; in comparison, when the humidity was 75%, the contact angle reached the maximum. With the increase in temperature, cement hydration is promoted, leading to the formation of a rough structure on the specimen surface. Moreover, the hydrophobic agent undergoes a condensation reaction, which enhances both the strength and the contact angle. Under low-humidity conditions, the hydrophobic agent cannot fully hydrolyze, whereas excessively high humidity causes the loss of hydrophobic groups. Under the curing conditions of 75% humidity and 40 °C, the specimen exhibits optimal performance.
(2) The surface texture structure shows a marked influence on the contact angle and rolling angle of the specimen; with an increase in the mesh number, the contact angle increases, whereas the rolling angle decreases. At a mesh number of 200 mesh, the specimen exhibits a contact angle above 160° and a rolling angle below 10°, indicating superhydrophobic behavior. However, when the mesh number is 250 mesh, the change in contact angle of TE4A6 is relatively stable, and the rolling angle increases slightly.
(3) Characteristic peaks of -CH_3_ and -CH_2_ can be observed in the FTIR test under different curing conditions, indicating no change in the physical phases of the specimens. In the AFM test, it was observed that the undulations of the specimen surface varied at different texture scales: higher mesh numbers resulted in larger undulations, and the texture of the specimen surface increased and then decreased when the mesh number exceeded 100 mesh (0.18 mm).

Optimizing curing methods can provide guidance for the construction of practical engineering projects. More efficient curing methods can enhance the performance of superhydrophobic concrete, extend its service life, reduce its lifecycle cost, and promote the green development of buildings.

In future research, we aim to investigate the performance variations of superhydrophobic concrete under wider temperature ranges. Combined with hydrodynamics, we will analyze the hydration products of superhydrophobic concrete and their roles in contributing to superhydrophobic properties, thereby conducting a more in-depth study. Meanwhile, corresponding research will be carried out on the dispersion methods and duration of silane, with the goal of optimizing the preparation process of superhydrophobic concrete in conjunction with practical engineering contexts.

## Figures and Tables

**Table 1:** Correspondence between mesh size and hole diameter.

Mesh Sizes	20	40	60	80	100	150	200	250
wire diameter (mm)	0.23	0.15	0.12	0.10	0.08	0.06	0.05	0.04
diameter of hole (mm)	1.05	0.48	0.30	0.21	0.18	0.11	0.08	0.05

**Table 2:** Mix proportion of concrete.

Sample	Cement	W/C	C/S	NS	DTMS	OTMS	TETPS	TIA	Superplasticizer
DT2A10	600	0.5	0.5	2%	2%	-	-	10%	1%
OT2A10	600	0.5	0.5	2%	-	2%	-	10%	1%
TE2A10	600	0.5	0.5	2%	-	-	2%	10%	1%
TE4A6	600	0.5	0.5	2%	-	-	4%	6%	1%

**Table 3:** Design of curing regimes.

Sample	Curing Humidity *	Curing Temperature **
DT2A10	35%/55%/75%/95%	10 °C/20 °C/30 °C/40 °C
OT2A10	35%/55%/75%/95%	10 °C/20 °C/30 °C/40 °C
TE2A10	35%/55%/75%/95%	10 °C/20 °C/30 °C/40 °C
TE4A6	35%/55%/75%/95%	10 °C/20 °C/30 °C/40 °C

* During curing humidity tests, the curing temperature was maintained at 30 °C. ** During curing temperature tests, the curing humidity was maintained at 75%.

**Table 4:** Compressive strength and contact angle of DT2A10 and OT2A10.

	Curing Humidity	Compressive Strength (MPa)	Contact Angle (°)
DT2A10	35%	9.4 (±0.2)	148.9 (±0.2)
	55%	9.2 (±0.5)	151.3 (±0.8)
	75%	12.0 (±0.7)	155.5 (±1.4)
	95%	22.3 (±0.8)	146.2 (±1.0)
OT2A10	35%	10.6 (±0.2)	149.5 (±0.6)
	55%	9.2 (±0.1)	154.3 (±0.7)
	75%	16.0 (±0.2)	156.3 (±1.6)
	95%	23.1 (±0.1)	146.3 (±1.2)

**Table 5:** Compressive strength and contact angle of TE4A6 and TE2A10.

	Curing Humidity	Compressive Strength (MPa)	Contact Angle (°)
TE4A6	35%	15.7 (±1.0)	151.7 (±1.4)
	55%	16.6 (±0.4)	155.2 (±1.2)
	75%	19.0 (±1.3)	158.8 (±0.9)
	95%	25.7 (±0.4)	146.7 (±1.3)
TE2A10	35%	11.6 (±0.2)	150.2 (±0.9)
	55%	12.7 (±0.6)	153.6 (±0.7)
	75%	16.5 (±0.6)	157.3 (±0.2)
	95%	23.9 (±1.0)	144.8 (±1.1)

**Table 6:** Compressive strength and contact angle of DT2A10 and OT2A10.

	Curing Temperature	Compressive Strength (MPa)	Contact Angle (°)
DT2A10	10 °C	3.7 (±0.3)	151.3 (±1.6)
	20 °C	14.1 (±0.6)	156.0 (±0.9)
	30 °C	15.6 (±1.2)	161.0 (±1.1)
	40 °C	19.9 (±0.5)	162.9 (±1.1)
OT2A10	10 °C	4.8 (±0.3)	154.2 (±1.2)
	20 °C	16.0 (±0.4)	157.5 (±1.1)
	30 °C	19.8 (±0.7)	161.3 (±1.3)
	40 °C	22.3 (±1.7)	164.2 (±1.4)

**Table 7:** Compressive strength and contact angle of DT2A10 and OT2A10.

	Curing Temperature	Compressive Strength (MPa)	Contact Angle (°)
DT2A10	10 °C	6.9 (±0.5)	154.9 (±1.2)
	20 °C	19.1 (±1.3)	158.8 (±1.0)
	30 °C	21.2 (±0.9)	163.8 (±1.1)
	40 °C	25.1 (±0.5)	166.3 (±1.0)
OT2A10	10 °C	5.9 (±0.1)	154.7 (±1.0)
	20 °C	17.0 (±1.0)	157.6 (±0.9)
	30 °C	20.4 (±0.4)	161.3 (±0.6)
	40 °C	23.5 (±0.6)	165.3 (±0.8)

**Table 8:** Effect of surface texture structure on contact angle and rolling angle.

	Mesh Size	Contact Angle (°)	Sliding Angle (°)
TE4A6	20	151.2 (±1.2)	36.1 (±2.7)
	40	155.2 (±0.7)	32.6 (±1.8)
	60	158.8 (±0.2)	25.3 (±1.1)
	80	161.4 (±0.7)	20.8 (±1.5)
	100	164.6 (±1.3)	15.2 (±2.3)
	150	167.7 (±1.5)	11.1 (±0.8)
	200	169.0 (±1.2)	7.6 (±1.0)
	250	168.8 (±1.0)	9.7 (±0.9)
TE2A10	20	149.7 (±1.1)	45.0 (±2.0)
	40	153.8 (±1.6)	40.0 (±1.8)
	60	157.4 (±1.4)	30.0 (±1.6)
	80	159.8 (±0.6)	24.0 (±1.3)
	100	161.9 (±1.4)	17.4 (±1.0)
	150	164.1 (±0.9)	11.8 (±0.9)
	200	166.4 (±0.9)	9.0 (±0.5)
	250	165.7 (±1.4)	10.6 (±0.9)

## Data Availability

The original contributions presented in this study are included in the article. Further inquiries can be directed to the corresponding author.
